# Disparities in survival after hip fractures in Chile in patients above 60 years: the impact of the operating room management

**DOI:** 10.3389/frhs.2026.1554376

**Published:** 2026-03-13

**Authors:** Maximiliano Barahona, Omar Matus, Susana Mondschein

**Affiliations:** 1 Department of Orthopaedic Surgery, Hospital Clínico Universidad de Chile, Santiago, Chile; 2 Department of Engineering, Universidad Adolfo Ibáñez, Santiago, Chile; 3 Department of Industrial Engineering, Universidad de Chile, Santiago, Chile; 4 Complex Engineering Systems Institute, Santiago, Chile

**Keywords:** hip fracture, incidence, mortality, operative room management, survival analysis

## Abstract

**Background:**

Hip fractures among individuals aged 60 and older significantly impact health, leading to increased morbidity, dependency, and financial strain while also elevating mortality risks. Variation in trauma management across healthcare settings underscores the urgent need for comprehensive data analysis to improve patient outcomes.

**Methods:**

This study employs an observational design. It analysed national databases from 2012 to 2017 to assess hip fracture incidence and survival rates among Chileans aged 60 and older. It examined the impact of factors such as age, type of health insurance provider, access to surgical treatment, and hospital type on mortality rates, using Kaplan–Meier and Cox proportional hazards models.

**Findings:**

The incidence of hip fractures in Chile remained constant, with the majority of cases being managed in public healthcare institutions. Public hospitals had longer stays and less access to surgery than private hospitals. Key mortality factors included access to surgery, age, previous hospitalisations, and gender, with survival rates lower than the general population, especially among patients treated in public hospitals.

**Interpretation:**

The findings of this study highlight the crucial need for public health initiatives that prioritise operating room management to improve surgical access and ensure timely care for patients with hip fractures. These findings provide policymakers in Chile with valuable insights to improve healthcare delivery and suggest that similar evaluations could benefit other countries with comparable demographics, potentially leading to significant improvements in healthcare policy.

## 1 Introduction

Hip fractures in individuals aged 60 years and older are associated with considerable health complications, increased dependency, financial burdens, and elevated mortality rates (1). These fractures represent one of the most severe and costly consequences of osteoporosis, often referred to as a silent epidemic (2). The organisation of health systems in managing trauma, particularly hip fractures, varies significantly across hospitals, cities, and countries, influencing the efficiency and effectiveness of care. Consequently, analysing large databases is essential for auditing care and implementing improvements that directly benefit patient outcomes (3).

A key indicator of the impact of hip fractures is the one-year mortality rate, defined as the proportion of patients who die from any cause within a year of the fracture. This rate is significantly affected by patients' comorbidities and the timeliness of surgical intervention (4). In countries such as the UK and Australia, healthcare systems have been optimised to promptly manage comorbidities and provide surgery within 36 h, demonstrating cost-effectiveness (4). For instance, in the UK, the Incremental Cost-Effectiveness Ratio (ICER) for hip fracture treatment during the first year aligns with the National Institute for Health and Care Excellence's cost-effectiveness threshold, ranging from 27,000 to 40,500 USD per Quality-Adjusted Life Year (QALY). By the second year, the ICER decreases to 11,900 USD per QALY (5). Similarly, in China, which has one of the lowest one-year mortality rates following hip fractures, performing surgery within 48 h of admission is cost-effective (6).

Chile's healthcare insurance system is a hybrid model with public and private providers. It includes: (i) FONASA, the public insurer covering 78% of the population; (ii) ISAPREs, private insurers covering 14%; and (iii) a system for the Military and Police Forces, serving 3%. FONASA beneficiaries are divided into four income-based tiers: “A” for those with no income, “B” for earners under 400,000 pesos (approx. 439 USD), “C” for those earning up to 584,000 pesos (approx. 641 USD) with fewer than three dependents, and “D” for higher incomes. Importantly, FONASA patients treated in public hospitals face zero copayment at the point of care. In contrast, those in tiers B, C, and D may also access private hospitals, but with varying copayments depending on their tier. ISAPRE patients predominantly use private hospitals, although they may occasionally utilise public hospitals under special payment arrangements (7). ISAPRE patients predominantly use private hospitals, although they may occasionally utilise public hospitals under special payment arrangements.

Chile's public health system allocates operating rooms for gyneco-obstetric, emergency, and elective procedures (8). Gyneco-obstetric operating rooms are primarily dedicated to childbirth and related interventions. Emergency operating rooms manage cases from emergency services or complications in hospitalised patients, with limited capacity for treating fractures, except for open fractures. Both operating rooms function 24/7. In contrast, elective operating rooms, functioning from 8 a.m. to 5 p.m. on weekdays, are designated for scheduled surgeries, often addressing conditions on the surgical waiting list (9). However, in orthopaedics, elective operating rooms are frequently occupied by fracture cases, including hip fractures awaiting surgery, which delays traditional elective procedures such as arthroplasties (10). The underutilisation of emergency operating rooms for hip fractures contributes to prolonged treatment times and exacerbates the backlog of orthopaedic surgeries.

Healthcare inequity has long been a concern in Chile and became a central theme during the 2019 social protests, when citizens explicitly voiced concerns about the quality, accessibility, and timeliness of public healthcare services (11). These systemic inequities are particularly evident in the care of older adults, who often rely on the public sector for urgent and resource-intensive conditions such as hip fractures. In this context, access to timely surgical treatment for hip fractures varies substantially across institutions, with important consequences for survival and long-term functional outcomes. In 2017, the incidence of hip fractures in Chile was estimated at 40 cases per 100,000 people, with a projected 28% increase by 2030 (12, 13). Despite a broad consensus among Chilean hip surgeons regarding the importance of early surgical management (14), recent data indicate that 10% to 18% of patients within the national public network do not undergo surgery, and median waiting times reach six days (13, 15). However, the extent to which these disparities reflect underlying patient characteristics vs. organisational factors related to perioperative care—particularly operating room availability—has not been formally evaluated at a national level.

Robust public health policies require a comprehensive epidemiological understanding of the conditions they aim to address, particularly when organisational performance is a potential driver of outcomes (3, 4). In this regard, national administrative data provide a unique opportunity to examine health-system performance and organisational determinants of outcomes at scale, especially when linked to mortality records. Accordingly, this study aims to (i) characterise hip fractures among Chileans aged 60 years and older between 2012 and 2017 using a nationally stratified database by sex, region, age, insurance coverage, and hospital type, and (ii) estimate survival following hip fracture. We hypothesise that disparities in financial coverage and hospital type (public vs. private) significantly influence survival, particularly through differences in access to surgical treatment. Additionally, we hypothesise that individuals sustaining a hip fracture experience reduced survival compared with the general population of the same age group, with a more pronounced effect among those treated in public hospitals.

## 2 Materials and methods

### 2.1 Ethics statement

This study utilised publicly accessible data from the Chilean Ministry of Health, specifically from the Department of Statistics and Health Information. All data were anonymised to safeguard personal information, which led the ethics committee to grant a waiver for this study, eliminating the need for participant consent.

### 2.2 Data description, inclusion, and exclusion criteria

We used two open-access national databases for this research. The first was the national hospital discharge registry managed by the Chilean Ministry of Health, which records all inpatient discharges from public and private healthcare facilities between 2002 and 2020. This database comprises 39 variables, including encrypted patient ID, diagnoses, gender, age, insurance status, hospital type, residency, length of stay, and discharge status. Our focus was on patients discharged with a primary diagnosis of hip fracture (ICD-10 codes S72.0, S72.1, S72.2) between January 1, 2012, and December 31, 2017, to ensure at least 1 year of follow-up, using death records available through 2018.

We identified 35,520 patients and 41,543 discharge episodes, with an average hospitalisation rate of 1.12 per patient (standard deviation of 0.39). Approximately 11.5% of patients were hospitalised more than once. Multiple discharges within 90 days were treated as a single episode to account for hospital transfers, rehospitalisations, or postoperative care. If a patient received treatment at a private and public hospital for the same event, the treatment location was recorded as the hospital where the surgery occurred. If no surgery was performed, the treatment location was defined as the hospital with the longest stay.

To validate the accuracy of the national discharge registry, we cross-checked a sample of cases with patient-level databases obtained from the electronic health records of two large hospitals (one public and one private). We matched cases based on admission and discharge dates, age, and sex, and specifically reviewed episodes that appeared in the national database but were not present in the hospital-level registry. This validation revealed that episodes recorded with a hip fracture diagnosis but surgical codes unrelated to hip fracture represented a systematic administrative error. These cases could therefore be confidently excluded, resulting in the removal of 4,455 observations. The list of included surgery codes is provided in Table 1.

**Table 1:** Procedural codes used to identify surgical management of hip fractures in Chilean hospital discharge data, 2012–2017.

Surgical Code	Description
2104128	Partial hip replacement with or without cementation (any technique) (does not include prosthetics)
2104129	Total hip replacement (does not include prosthesis)
2104131	Fracture of the femoral neck, osteosynthesis, any technique (does not include osteosynthesis elements).
2104132	Fracture of the femoral neck, femoral epiphysis resection
2104135	Salvage of the hip
2104228	Partial hip replacement with or without cementation (any technique) (includes prosthetics)
2104229	Total hip replacement (includes prosthesis)
2104231	Fracture of the femoral neck, osteosynthesis, or any technique (including osteosynthesis elements).

List of surgical procedures and corresponding national health classification codes (DEIS–MINSAL). These codes were used to categorise patients as having received either operative or non-operative treatment following hospital admission for a hip fracture.

In contrast, for patients with a primary diagnosis of hip fracture but no associated surgical procedure, we did not identify a systematic coding pattern that could explain their classification. Consequently, these cases were retained in the database as non-surgical treatments for hip fractures. As missing data represented less than 2% of the study population, we used a complete-case approach. Only hospital discharges with missing or inconsistent essential variables were removed—specifically, 388 cases without patient identifiers and 4 with conflicting demographic information.

In this dataset, cases classified as “non-surgical treatment” do not represent a defined therapeutic modality but rather the absence of any surgical procedure associated with the hospital discharge record; this classification reflects a database-related limitation rather than a clinical decision category. Consequently, patients without a surgical code were categorised as non-surgical. This group may include individuals who died before undergoing surgery, those with rare fracture patterns managed conservatively, or cases of administrative miscoding. However, the national database does not provide information on the clinical or organisational reasons for the absence of surgery. Therefore, it is not possible to determine whether a patient was not operated on because they died shortly after admission or died while awaiting surgery, because surgery was medically contraindicated or postponed due to clinical instability, or because of delays in operating room availability leading to non-surgical management. Given this limitation and the lack of evidence of a systematic coding error after validation against hospital-level records, these cases were retained in the analysis, and no further stratification was feasible. As the database does not provide evidence of differential miscoding between sectors, we assumed that any potential misclassification is homogeneously distributed across public and private institutions, which would bias the results towards the null rather than exaggerate differences.

The second database, managed by the Chilean Ministry of Health, records all deaths in Chile, including causes and associated diagnoses. At the time of the study, it included publicly available records of individuals who had died by December 31, 2018. For our research, we extracted records from January 1, 2012, to December 31, 2018, ensuring a minimum of one year of follow-up for all patients in the hospital discharge database. Both databases utilise consistent, encrypted patient IDs, enabling the accurate tracking of patient outcomes and prior hospitalisations.

### 2.3 Incidence & mortality rates

We compared total, crude, and age-standardised incidence rates per 100,000 inhabitants using the Segi World standard population (16). Incidence rates were computed as the number of cases per 100,000 inhabitants in the corresponding group under analysis. In the context of mortality, define *n*-year mortality rate as the fraction of hip-fracture patients who have died by the $n^{th}\;$ year. Analysis was conducted for the entire population and separately by gender, health insurance provider (public vs. private), region, and age groups: 60–69, 70–79, and 80 and above.

The general population dataset was obtained from the National Socioeconomic Characterisation Survey (17), which provides a detailed breakdown by gender, health insurance type, and region. Additionally, mortality data for the general population were sourced from the death registry maintained by the Chilean Institute of Statistics in Health.

### 2.4 Survival analysis

The primary outcome was the time from the initial hip fracture diagnosis to death, as recorded in the death database. Patients not listed in the death database by the end of the study (December 31, 2018) were considered survivors, with their deaths right-censored.

The variables analysed for post-hip fracture mortality risk in this study include age (discrete-continuous variable, starting at 60), sex, type of health insurance (public or private), hospital type (public or private), year of hospital discharge (2012–2017), number of transfers during the same episode (discrete-continuous variable), length of hospital stay (discrete-continuous), access to surgical treatment (yes or no), number of prior hospitalisations (discrete-continuous), duration of previous hospitalisations, and region of residence in Chile (categorical, I to XVI regions). The national database does not record either the exact time from admission to surgery or detailed clinical severity measures such as the ASA score or Charlson Comorbidity Index; therefore, length of hospital stay was used as an indirect proxy for perioperative delay, and prior hospitalisations—particularly for cardiovascular- or tumour-related conditions—were included as a pragmatic proxy for comorbidity burden.

We created two additional variables for this study. The first combined the type of hospital and health insurance provider into 10 categories, based on the five insurance types (private and four public tiers) and the two hospital types (public or private). The second variable examined patients' previous hospitalisations in the five years before their hip fracture, categorised into five groups: (i) no previous hospitalisations (w/o PH), (ii) previous hospitalisations unrelated to tumors or cardiovascular diagnoses (PH unrelated to T or C), (iii) hospitalisations with cardiovascular-related diagnoses (PH with C & w/o T), (iv) hospitalisations with tumor-related diagnoses (PH with T & w/o C), and (v) hospitalisations with both diagnoses (PH with C & T).

We used the Kaplan–Meier estimator with 95% confidence intervals for descriptive survival analysis. Subsequently, a multivariable Cox proportional hazards regression model was applied to identify significant risk factors for post–hip fracture mortality, testing all previously described variables. Variable selection was performed using a stepwise strategy guided by the Akaike Information Criterion (AIC): starting with the most significant covariate and sequentially adding those that reduced the AIC and presented a significant *p*-value (<0.05). Variables that did not improve model fit or violated selection criteria were excluded from the final model. The proportional hazards assumption was assessed using Schoenfeld residuals for each covariate and globally. Model discrimination was evaluated using Harrell's C statistic.

For categorical variables where no single category is more relevant, such as gender, we included the category with better survival in the model's intercept. It is essential to note that the selected category does not affect the results, only their interpretation, and was used to facilitate the understanding of the hazard ratio. Consequently, the intercept included the female gender, the ISAPRE-private hospital combination, and no previous hospitalisations. The only exception was the patient's region of residence, where the Metropolitan Region (Region XIII) was used as the intercept due to its significantly larger population than other regions in Chile.

All data processing and statistical analyses were conducted using R (v 4.2.2, The R Foundation for Statistical Computing, Vienna, Austria) within RStudio (version 2023.03.0, RStudio, PBC, Boston, MA). Survival analysis was performed using the survival analysis package (version 3.6-4).

## 3 Results

### 3.1 Descriptive statistics

During the study period, a total of 33,132 hip fractures were recorded among individuals aged 60 and older. Table 2 summarises the main characteristics of these patients. The average age exceeded 80 years, and women consistently represented more than 75% of cases. FONASA covered most patients (over 85%), with nearly 90% of them receiving treatment in public hospitals. More than 20% of patients received non-surgical treatment each year, particularly in public institutions. Hospital stays in public facilities were markedly longer—averaging 18.9 days—compared with 10.9 days in private hospitals. (see also Supplementary Table S1).

**Table 2:** Annual number of hip fractures, surgical management, sex distribution, and average hospital stay among Chilean patients aged ≥60 years, 2012–2017.

Variable	2012	2013	2014	2015	2016	2017	Total
Number of hip fractures	5,179	5,206	5,156	5,592	5,803	6,196	33,132
Number of hip surgeries (% surgeries from total hip fractures)	3,896 (75%)	3,885 (75%)	3,944 (76%)	4,280 (77%)	4,475 (77%)	4,813 (78%)	25,293 (76%)
Sex							
Male hip fractures (%)	1,161 (22%)	1,228 (24%)	1,172 (23%)	1,249 (22%)	1,349 (23%)	1,375 (22%)	7,534 (23%)
Female hip fractures (%)	4,018 (78%)	3,978 (76%)	3,984 (77%)	4,343 (78%)	4,454 (77%)	4,821 (78%)	25,598 (77%)
Length of hospital stay in days (standard deviation)							
Overall	17.9 (17.7)	18.1 (17.7)	17.8 (16.0)	18.4 (19.0)	17.3 (17.2)	16.7 (17.6)	17.7 (17.6)
Public hospitals	19.1 (18.5)	19.2 (18.3)	18.9 (16.4)	19.7 (19.8)	18.6 (18.0)	18.0 (18.5)	18.9 (18.3)
Private hospitals	11.1 (9.7)	11.3 (10.8)	11.5 (11.3)	11.0 (11.3)	10.8 (10.6)	10.0 (8.9)	10.9 (10.4)

Descriptive statistics of hip fracture hospitalisations, showing total cases, proportion undergoing surgery, sex distribution, and mean length of hospital stay (overall and by hospital type). Values in parentheses represent standard deviations

FONASA = Chile's public health insurer. **FONASA care delivered in public hospitals has zero copayment**; when FONASA beneficiaries access private hospitals under institutional agreements, copayments vary by tier (B–D). ISAPRE = private insurers with plan-specific deductibles/copayments.

“Non-surgical treatment” refers to patients with a **primary diagnosis of hip fracture who do not have any associated surgical procedure code** in the national database. This group may include rare atypical fractures or intrahospital deaths before surgery. However, validation against hospital-level data and regional evidence (Uruguayan study) suggests that such cases account for **6% of non-operated patients**, indicating that most reflect the actual absence of surgery.

### 3.2 Hip fracture incidence and mortality analysis

**Figure 1:**
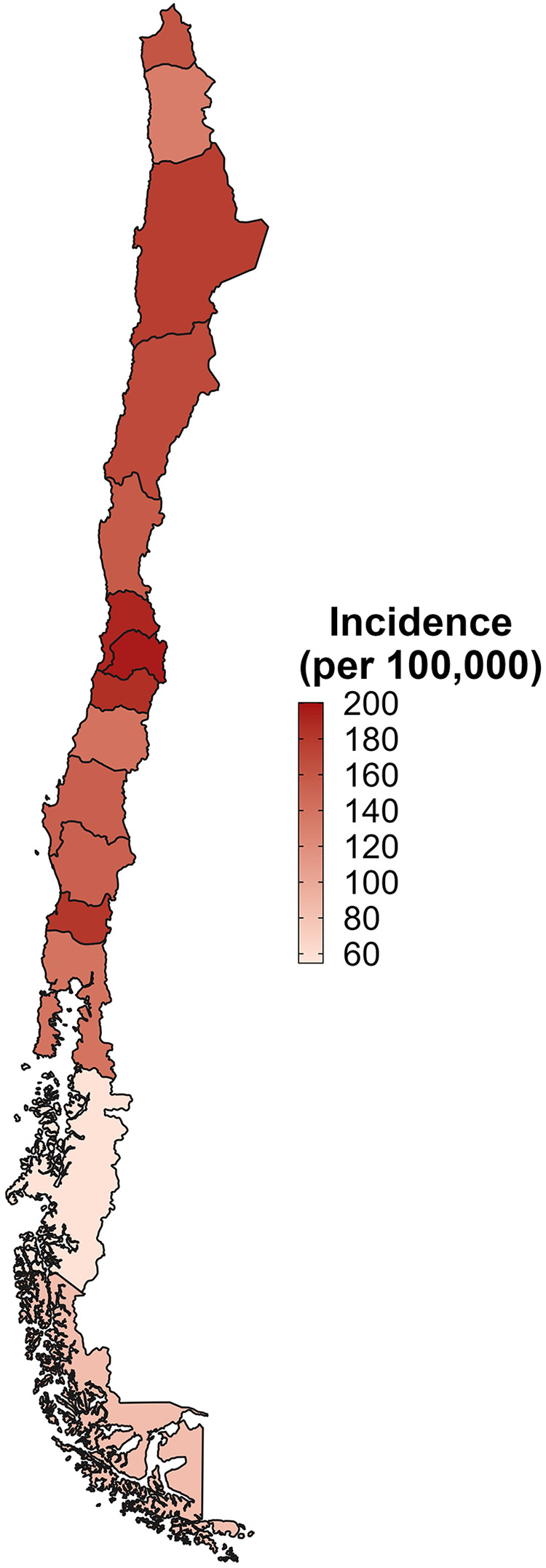
Geographic distribution of hip fracture incidence among adults aged ≥60 years in Chile, 2012–2017. Age-adjusted incidence rates of hip fracture per 100,000 inhabitants are shown by region. A higher incidence is observed in the northern and central regions, particularly in the Metropolitan, Valparaíso, and O'Higgins areas, while the southern regions exhibit lower rates. Colour intensity represents the magnitude of the incident light.

The crude incidence rate of hip fractures in Chile among individuals aged 60 and older ranged from 187.5 to 172.7 per 100,000 inhabitants, showing no upward trend. In contrast, the age-adjusted incidence for this demographic declined consistently, from 161.6 in 2012 to 144.3 in 2017 (Table 3). Analysis by age group revealed the highest incidence rates in those over 80, peaking at 693.0 cases per 100,000 in 2012. The 60- to 69-year-old and 70- to 79-year-old age groups also recorded their highest incidence rates in 2012, at 38.7 and 158.6 cases, respectively (Table 3). Throughout the years, incidence rates were higher among female patients and those with public health insurance (FONASA) (Table 3). Geographically, incidence rates varied across the country, with the highest values observed in the Metropolitan (XIII), Valparaíso (V), and O'Higgins (VI) regions (Figure 1).

**Table 3:** Crude and age-adjusted incidence rates of hip fracture among Chilean adults aged ≥60 years, 2012–2017.

Incidence	2012	2013	2014	2015	2016	2017
Crude 60+	187.5	176.3	167.7	176.5	171.2	172.7
Age-Adjusted 60+	161.6	150.5	140.8	147.8	142.3	144.3
Crude by age						
60–69 years	38.7	36.9	35.6	36.3	34.3	35.2
70–79 years	158.6	145.0	138.7	145.6	134.4	137.6
80+ years	693.0	663.9	624.7	654.1	659.8	665.8
Crude 60+						
Male	99.3	97.6	89.1	92.4	92.5	88.2
Female	252.3	234.9	226.3	239.2	230.6	237.3
FONASA	191.6	183.0	173.6	182.1	176.2	178.9
ISAPRE	122.7	103.6	107.0	126.3	115.4	124.5

Annual crude and age-adjusted incidence rates per 100,000 inhabitants are presented for the total population aged 60 years and older, stratified by age group, sex, and type of health insurance (FONASA or ISAPRE). Rates were calculated using national hospital discharge records and population estimates from the National Institute of Statistics (INE).

**Figure 2:**
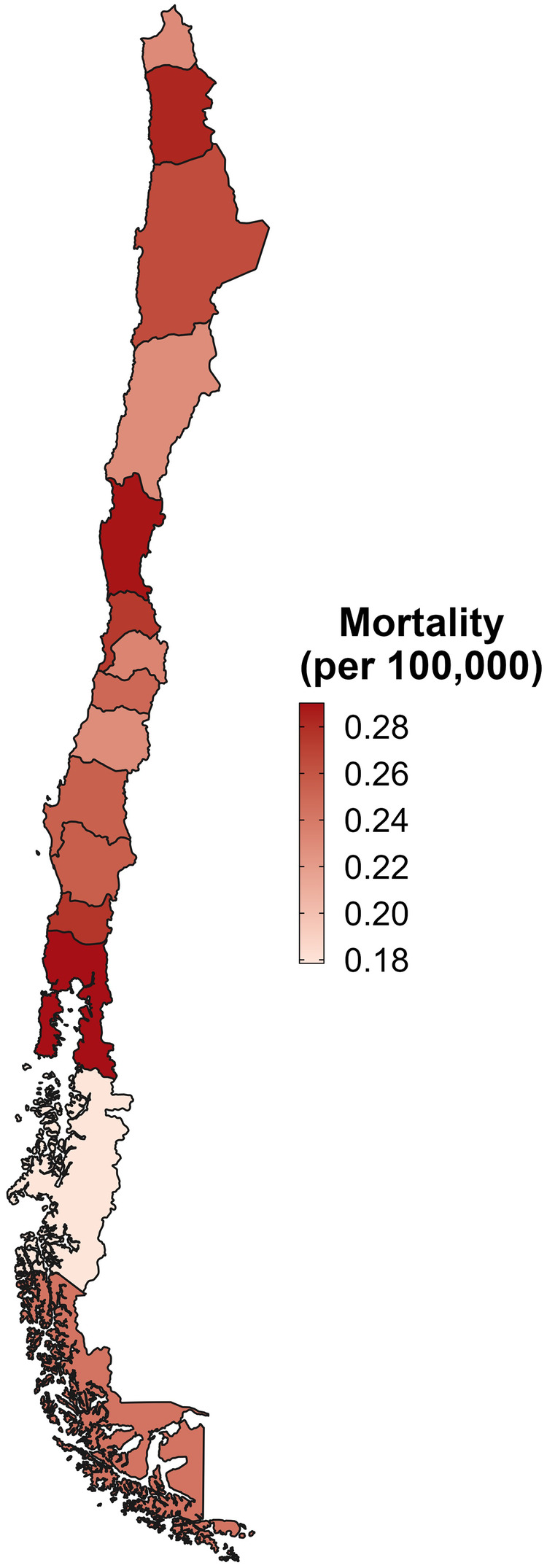
Geographic distribution of one-year mortality following hip fracture among adults aged ≥60 years in Chile, 2012–2017. Age-adjusted one-year mortality rates per 100,000 inhabitants are shown by region. Higher mortality is observed in the northern (Antofagasta, II Region) and southern (Los Lagos, X Region) areas, with intermediate levels across central Chile. Darker shading represents higher mortality.

The one-year mortality rate following hip fracture remained relatively stable from 2012 to 2017, decreasing slightly from 25.7% in 2012 to 24.5% in 2017 (Table 4). Individuals over 80 consistently exhibited the highest mortality rates, while men had higher one-year mortality rates than women, with ratios ranging from 1.3 in 2016 to 1.4 in 2017. Patients treated in public hospitals showed higher mortality rates than those in private hospitals, with a peak ratio of 2.2 in 2016 and a low of 1.6 in 2012 (Table 4). Geographically, mortality rates showed a heterogeneous distribution, with the highest values observed in Antofagasta (II), Coquimbo (IV), and Los Lagos (X) regions. Unlike incidence, which was concentrated in contiguous central regions, the areas with the highest mortality were geographically distant, suggesting that regional disparities in healthcare access and patient characteristics may influence post-fracture survival (Figure 2).

**Table 4:** One-year mortality rates after hip fracture among Chilean adults aged ≥60 years, 2012–2017.

One-year mortality rate	2012	2013	2014	2015	2016	2017
60+	0.257	0.259	0.248	0.251	0.242	0.245
One-year mortality rate by age						
60–69 years	0.116	0.139	0.149	0.132	0.132	0.136
70–79 years	0.189	0.203	0.183	0.176	0.171	0.164
80+ years	0.309	0.302	0.292	0.302	0.287	0.294
One-year mortality rate by gender for patients 60+						
Male	0.326	0.325	0.311	0.307	0.301	0.316
Female	0.237	0.238	0.229	0.236	0.225	0.224
One-year mortality rate by type of hospital for patients 60+						
Public hospital	0.265	0.263	0.260	0.259	0.250	0.254
Private hospital	0.164	0.155	0.137	0.157	0.113	0.147
One-year mortality rate by type of healthcare insurance provider for patients 60+						
Fonasa	0.247	0.243	0.244	0.244	0.233	0.240
Isapre	0.144	0.133	0.114	0.138	0.101	0.131
One-year mortality rate by type of previous hospitalisation (PH) for patients 60+						
w/o PH	0.231	0.226	0.231	0.228	0.228	0.223
PH unrelated to T or C	0.246	0.253	0.250	0.247	0.234	0.241
PH with C w/o T	0.325	0.317	0.299	0.300	0.265	0.298
PH with T w/o C	0.374	0.335	0.313	0.309	0.291	0.325
PH with C & T	0.356	0.357	0.319	0.275	0.468	0.325

Annual one-year mortality rates are presented overall and stratified by age group, sex, hospital type (public or private), insurance type (FONASA or ISAPRE), and history of previous hospitalisation (PH) related to cardiovascular (C) or tumoral (T) conditions.

Regarding comorbidities, patients without prior hospitalisations (No PH) had the lowest one-year mortality rate, followed by those hospitalised for conditions unrelated to tumours or cardiovascular diseases (PH unrelated to T or C). In contrast, individuals with hospitalisations for cardiovascular or tumoral reasons faced a significantly higher one-year mortality rate, nearing 30% (Table 4).

**Figure 3:**
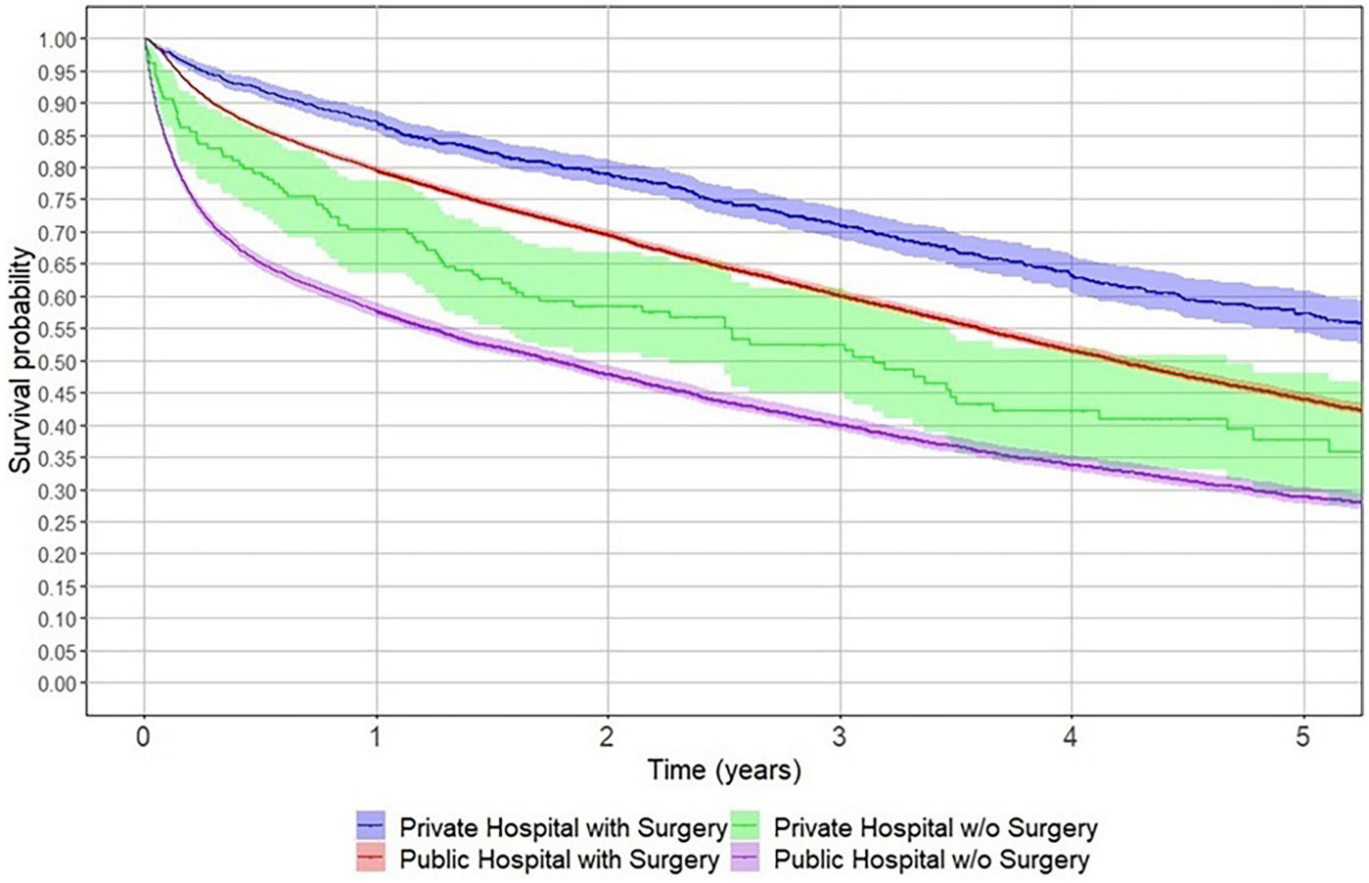
Five-year survival of older adults with hip fracture by hospital type and surgical treatment in Chile, 2012–2017. Kaplan–Meier survival curves display the probability of survival after hip fracture among patients aged 60 years or older, stratified by hospital type (public or private) and surgical management (with or without surgery). Survival was highest among patients treated surgically in private hospitals and lowest among those managed without surgery in public hospitals. Shaded bands represent 95% confidence intervals.

**Figure 4:**
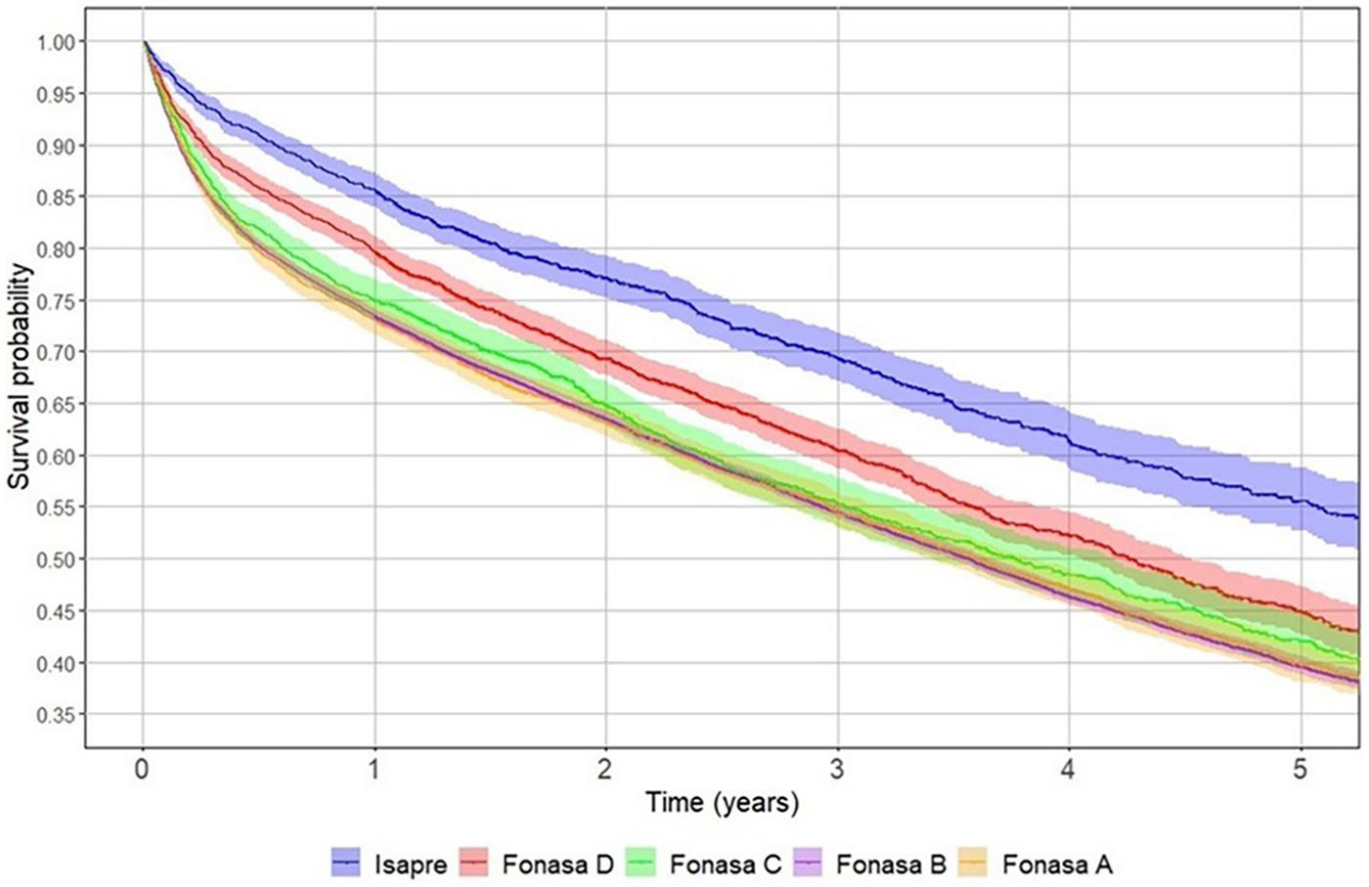
Five-year survival after hip fracture by health insurance category in Chile, 2012–2017. Kaplan–Meier survival curves compare survival probabilities among adults aged 60 years or older hospitalised for hip fracture, stratified by health insurance: public insurance (FONASA groups A–D) and private insurance (ISAPRE). Patients with private insurance showed consistently higher survival than those covered under FONASA, with the poorest outcomes observed in FONASA A. Shaded bands represent 95% confidence intervals.

**Figure 5:**
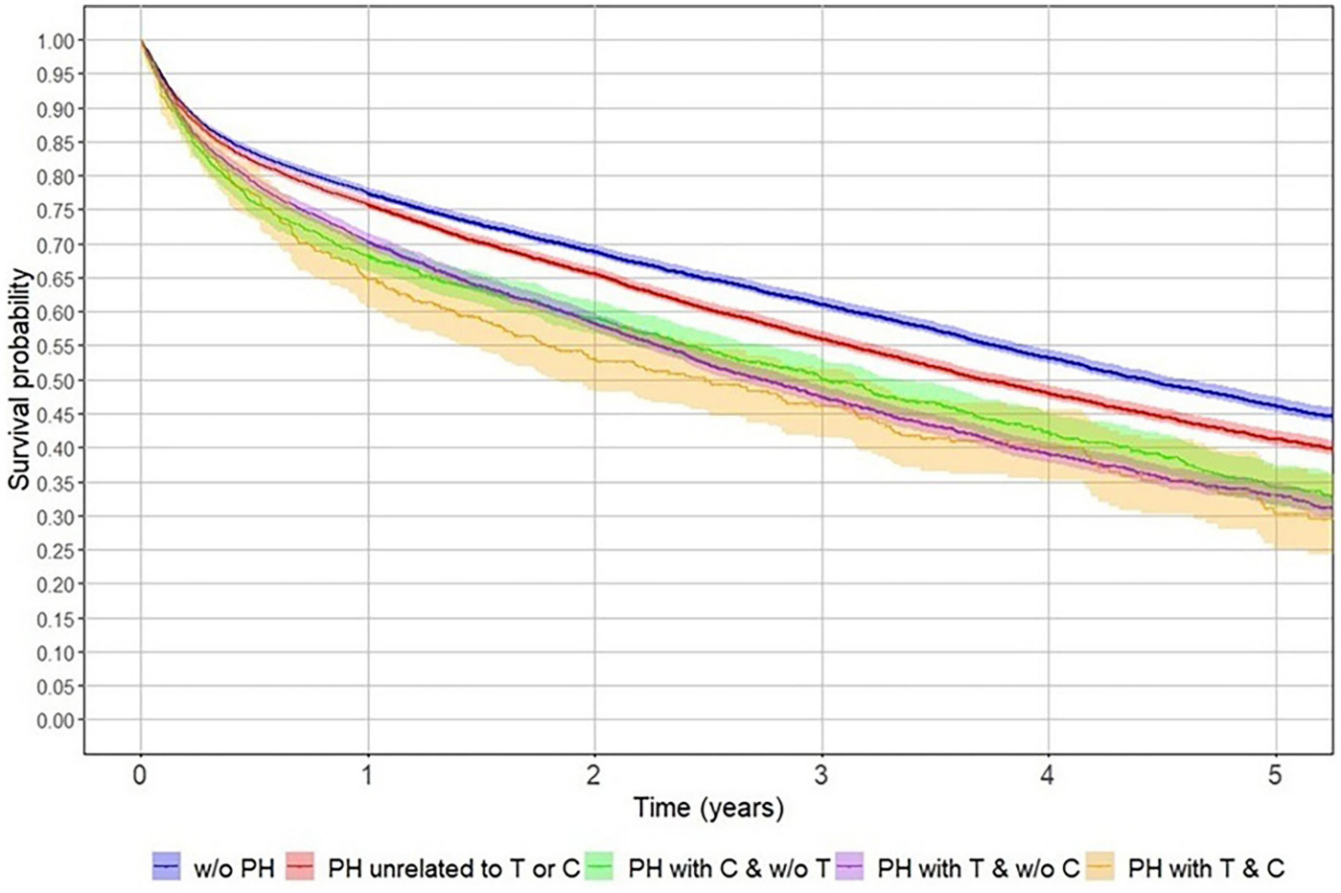
Impact of prior hospitalisation on post–hip fracture survival in Chile, 2012–2017. Kaplan–Meier survival curves show the five-year survival of patients aged ≥60 years according to history of previous hospitalisation (PH) related to tumoral (T) or cardiovascular (C) conditions. Patients with prior tumoral hospitalisations exhibited the lowest survival after hip fracture, followed by those with cardiovascular-related PH, whereas individuals without previous hospitalisations showed the best outcomes. Shaded bands represent 95% confidence intervals.

Undergoing surgery, regardless of hospital type (private or public), significantly improved five-year survival rates (Figure 3). Patients with private health insurance had better survival rates than those with public insurance. Beneficiaries in the highest tier (FONASA D) had the best survival rates among public insurance holders (Figure 4). Additionally, patients with prior hospitalisations for tumours or cardiac disease had lower five-year survival rates than those hospitalised for other reasons or those without prior hospitalisations (Figure 5).

The multivariate Cox proportional hazards model identified several significant factors associated with reduced survival following a hip fracture (Table 5). These factors include age (Hazard Ratio [HR] = 1.06), lack of surgical treatment (HR = 1.72), male gender (HR = 1.53), and length of hospital stay (HR = 1.004) (Table 5). Regardless of insurance type, patients treated in public hospitals had consistently lower survival rates than those in ISAPRE-private hospitals, particularly among lower FONASA tiers. ISAPRE patients treated in public hospitals were rare (4%), resulting in wide confidence intervals. The analysis revealed no significant impact on survival rates for patients who combined FONASA tiers with treatment in private hospitals.

**Table 5:** Multivariable Cox proportional hazards model for survival after hip fracture among Chilean adults aged ≥60 years, 2012–2017.

Variable	Coef	HR	Pr(>|z|)
Age	0.058	1.06 (1.06–1.06)	2 × 10^−16^
No surgery	0.541	1.72 (1.66–1.78)	2 × 10^−16^
Male sex	0.423	1.53 (1.47–1.58)	2 × 10^−16^
Length of hospital stay (days)	0.004	1.004 (1.003–1.004)	2 × 10^−16^
Public hospital/Isapre	0.695	2.01 (1.61–2.50)	7.01 × 10^−10^
Public hospital/Fonasa A	0.528	1.70 (1.57–1.83)	2 × 10^−16^
Public/hospital Fonasa B	0.532	1.70 (1.60–1.81)	2 × 10^−16^
Public hospital/Fonasa C	0.466	1.59 (1.46–1.74)	2 × 10^−16^
Public hospital/Fonasa D	0.412	1.51 (1.38–1.65)	2 × 10^−16^
Region I	0.276	1.32 (1.13–1.53)	0.0003
Region VII	−0.094	0.91 (0.85–0.98)	0.01140
Region VIII	−0.065	0.94 (0.88–0.99)	0.03140
Region IX	−0.153	0.86 (0.80–0.92)	0.00003
PH not C or T related	0.079	1.08 (1.07–1.10)	2 × 10^−16^
C PH	0.166	1.18 (1.15–1.22)	2 × 10^−16^
T PH	0.277	1.32 (1.24–1.40)	2 × 10^−16^

Hazard ratios (HR) with 95% confidence intervals (CI) are shown for age, sex, insurance type, hospital type, surgical treatment, length of hospital stay, geographic region, and history of previous hospitalisation. Reference categories: female sex, ISAPRE/private hospital with surgery, no previous hospitalisation, and Metropolitan Region. PH, previous hospitalisation; C, cardiovascular; T, tumoral.

Regarding prior hospitalisations, those unrelated to tumours or cardiovascular issues were associated with a modest but significant reduction in survival (HR = 1.08) compared to patients without previous hospitalisations. More pronounced reductions in survival were observed for hospitalisations related to cardiovascular conditions (HR = 1.18) and tumours (HR = 1.32), both of which significantly impacted five-year survival. Additionally, survival rates were higher for patients treated in the Tarapacá, Maule, Biobío, and Araucanía regions than those treated in the Metropolitan Region (Table 5).

Variables such as “year of hospital discharge”, “number of transfers for the same episode”, and “number of previous hospitalisations” did not reach statistical significance and were thus excluded from the model. Furthermore, the proportional hazards assumption was not violated (*p*-value > 0.10), and the model demonstrated moderate discriminative ability (Harrell's C = 0.7).

**Figure 6:**
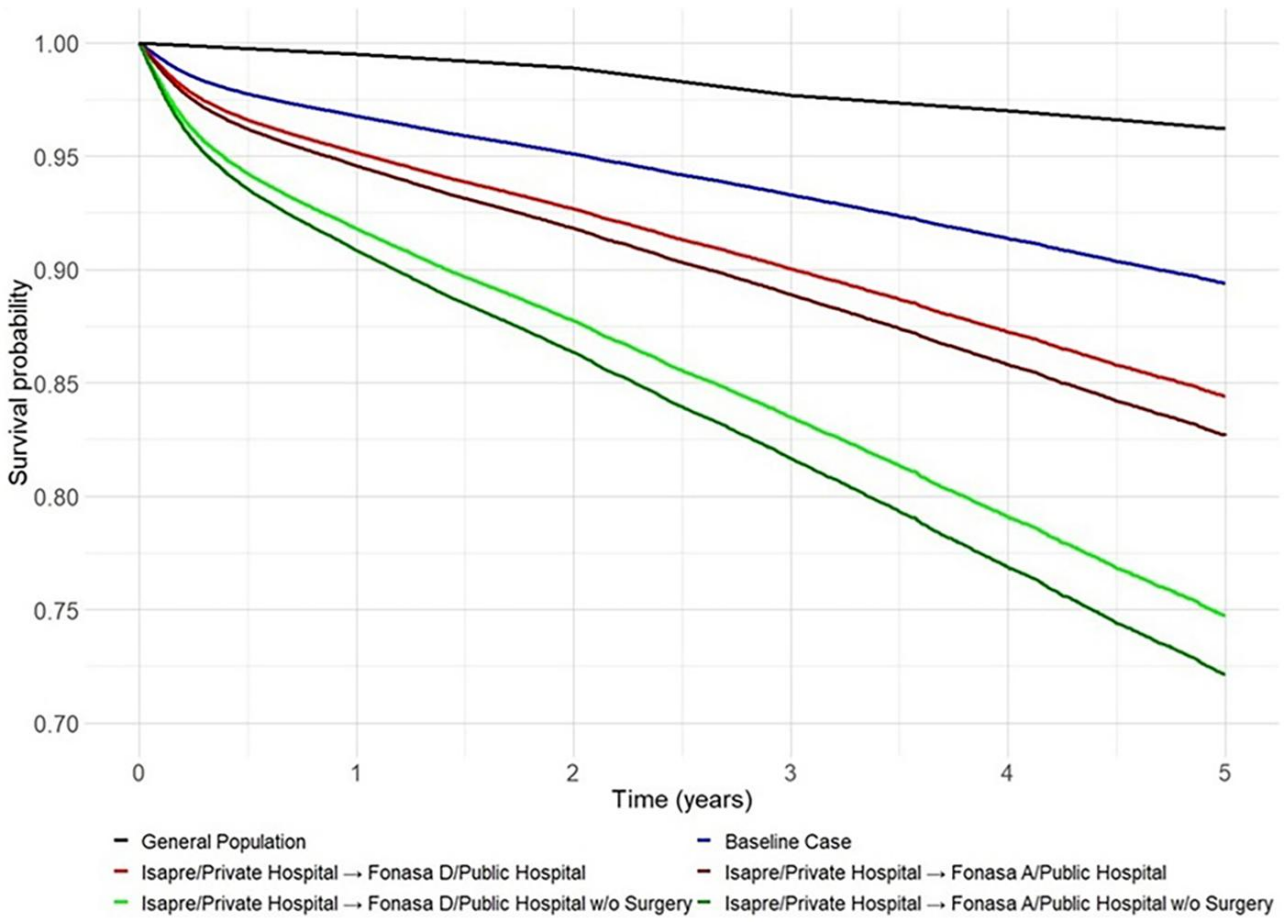
Predicted five-year survival under different policy scenarios combining health insurance, hospital type, and surgical treatment in Chile, 2012–2017. Predictions are derived from a Cox proportional hazards model fitted to patients aged 60 years or older with hip fractures. The baseline corresponds to a 60-year-old woman with ISAPRE coverage, treated surgically in a private hospital with a 17-day hospital stay. Comparative scenarios illustrate the impact on survival of transitioning to public insurance (FONASA A or D), receiving care in public hospitals, and non-surgical treatment. The curve for the general population represents the expected survival of individuals aged 60–64 years without a hip fracture.

According to the model, female patients aged 60, those with private insurance, individuals who underwent surgery, those treated in private facilities, and patients without prior hospitalisations in the last five years constitute the combination of factors associated with the best chance of survival following a hip fracture. However, despite representing the best outcome scenario, the estimated survival rate for these patients is still lower than that of the general population of the same age range (60–64) and gender (female) (Figure 6). Table 6 provides a detailed estimation of one-year mortality using the Cox model for patients aged 60, 70, and 80 years, considering various combinations of sex, type of surgery, length of hospital stays, comorbidities, insurance provider, and hospital.

**Table 6:** Model-based predicted one-year mortality after hip fracture by demographic and treatment characteristics in Chile, 2012–2017.

Insurance-hospital type	Age	60								70								80							
	Sex	Male				Female				Male				Female				Male				Female			
	Surgery	No		Yes		No		Yes		No		Yes		No		Yes		No		Yes		No		Yes	
	Hospital Stay (days) PH	10	15	10	15	10	15	10	15	10	15	10	15	10	15	10	15	10	15	10	15	10	15	10	15
Isapre Private Hospital	w/o PH	92%	92%	95%	95%	95%	95%	97%	97%	86%	86%	92%	91%	91%	90%	94%	94%	76%	76%	86%	85%	84%	84%	90%	90%
	PH unrelated C&T	91%	91%	95%	95%	94%	94%	97%	97%	85%	85%	91%	91%	90%	90%	94%	94%	75%	74%	84%	84%	83%	82%	90%	89%
	PH w/ T	89%	89%	94%	94%	93%	93%	96%	96%	82%	82%	89%	89%	88%	88%	93%	93%	70%	70%	81%	81%	79%	79%	87%	87%
	PH w/ C	91%	90%	94%	94%	94%	94%	96%	96%	84%	83%	90%	90%	89%	89%	93%	93%	73%	72%	83%	83%	81%	81%	89%	88%
Isapre Public Hospital	w/o PH	84%	84%	91%	90%	89%	89%	94%	94%	74%	73%	84%	84%	82%	82%	89%	89%	58%	58%	73%	73%	70%	70%	81%	81%
	PH unrelated C&T	83%	83%	90%	90%	89%	88%	93%	93%	72%	72%	83%	82%	81%	80%	88%	88%	56%	55%	71%	71%	68%	68%	80%	80%
	PH w/ T	80%	80%	88%	88%	86%	86%	92%	92%	67%	67%	79%	79%	77%	77%	86%	86%	49%	48%	66%	65%	63%	62%	76%	76%
	PH w/ C	82%	82%	89%	89%	88%	87%	93%	93%	70%	69%	81%	81%	79%	79%	87%	87%	53%	52%	69%	68%	66%	65%	78%	78%
FONASA D Public Hospital	w/o PH	88%	88%	93%	93%	92%	92%	95%	95%	80%	79%	88%	87%	86%	86%	92%	92%	67%	66%	79%	79%	77%	76%	86%	85%
	PH unrelated C&T	87%	87%	92%	92%	91%	91%	95%	95%	78%	78%	87%	86%	85%	85%	91%	91%	64%	64%	77%	77%	75%	75%	85%	84%
	PH w/ T	85%	84%	91%	91%	90%	89%	94%	94%	74%	74%	84%	84%	82%	82%	89%	89%	59%	58%	73%	73%	70%	70%	82%	81%
	PH w/ C	86%	86%	92%	91%	91%	90%	94%	94%	76%	76%	86%	85%	84%	84%	90%	90%	62%	61%	76%	75%	73%	73%	83%	83%
FONASA C Public Hospital	w/o PH	87%	87%	92%	92%	92%	91%	95%	95%	79%	78%	87%	87%	85%	85%	91%	91%	65%	65%	78%	78%	76%	75%	85%	85%
	PH unrelated C&T	86%	86%	92%	92%	91%	91%	95%	95%	77%	77%	86%	86%	84%	84%	91%	90%	63%	62%	76%	76%	74%	73%	84%	84%
	PH w/ T	84%	83%	90%	90%	89%	89%	93%	93%	73%	72%	83%	83%	81%	81%	89%	88%	57%	56%	72%	72%	69%	69%	81%	80%
	PH w/ C	85%	85%	91%	91%	90%	90%	94%	94%	75%	75%	85%	85%	83%	83%	90%	90%	60%	60%	75%	74%	72%	71%	82%	82%
FONASA B Public Hospital	w/o PH	87%	86%	92%	92%	91%	91%	95%	95%	77%	77%	86%	86%	85%	84%	91%	91%	63%	63%	77%	76%	74%	74%	84%	84%
	PH unrelated C&T	86%	85%	91%	91%	90%	90%	94%	94%	76%	75%	85%	85%	83%	83%	90%	90%	61%	60%	75%	75%	72%	72%	83%	83%
	PH w/ T	83%	82%	90%	89%	88%	88%	93%	93%	71%	71%	82%	82%	80%	80%	88%	88%	55%	54%	70%	70%	67%	67%	79%	79%
	PH w/ C	84%	84%	91%	90%	90%	89%	94%	94%	74%	74%	84%	84%	82%	82%	89%	89%	58%	58%	73%	73%	70%	70%	81%	81%
FONASA A Public Hospital	w/o PH	87%	86%	92%	92%	91%	91%	95%	95%	78%	77%	86%	86%	85%	84%	91%	91%	63%	63%	77%	76%	74%	74%	84%	84%
	PH unrelated C&T	86%	85%	91%	91%	90%	90%	94%	94%	76%	76%	85%	85%	83%	83%	90%	90%	61%	61%	75%	75%	72%	72%	83%	83%
	PH w/ T	83%	83%	90%	89%	88%	88%	93%	93%	71%	71%	82%	82%	80%	80%	88%	88%	55%	54%	70%	70%	67%	67%	80%	79%
	PH w/ C	85%	84%	91%	91%	90%	89%	94%	94%	74%	74%	84%	84%	82%	82%	89%	89%	58%	58%	73%	73%	70%	70%	81%	81%

Predicted probabilities of death within one year after hip fracture, derived from the multivariable Cox regression model, are shown for representative combinations of sex, surgical treatment, length of hospital stay, comorbidity type, insurance category, and hospital type. Estimates were calculated for patients aged 60, 70, and 80 years. PH, previous hospitalisation; C, cardiovascular; T, tumoral.

## 4 Discussion

Our study demonstrates that hip fractures are associated with a substantial excess risk of mortality compared with the general population of the same age and sex. As shown in Figure 4, even in the most favourable scenario according to the Cox model (female, age 60, ISAPRE coverage, treatment in a private hospital, and short length of stay), survival after hip fracture does not reach that of the general population, underscoring the profound and lasting impact of this condition. The Cox model also identified both modifiable and non-modifiable risk factors: age, male sex, and previous hospitalisations emerged as non-modifiable determinants of higher mortality. While age and male sex have been extensively studied (18–20), our findings highlight previous hospitalisations as an important marker of comorbidity severity that contributes significantly to excess mortality.

Beyond these non-modifiable determinants, our study also underscores the importance of modifiable risk factors, particularly those related to healthcare organisation. The most critical findings of our study are modifiable risk factors, including treatment in a public hospital, lack of surgical intervention, and prolonged hospital stays. Although these factors may seem distinct, they highlight a common challenge that is shared among them. Based on our findings**,** we recommend prioritising the optimisation of the care pathway for patients with hip fractures in the public healthcare system. This should be considered a critical policy intervention, given the high social and economic cost of delayed or absent surgical treatment in this population (21, 22).

Access to proper treatment is crucial for achieving favourable outcomes in hip fracture cases, specifically, timely surgery (23, 24). Our study reveals a concerning trend: over 20% of patients—especially those treated in public institutions—are not receiving surgical intervention. This rate is slightly higher than the 18% reported in a 2015 study conducted in Chile's second-most-populous city (15). Excessive conservative treatment may stem from health barriers. Still, it could also be influenced by specific types of hip fractures (e.g., isolated greater and lesser trochanter fractures), typically managed non-surgically (25, 26). Additionally, some fractures may be misclassified and not fit the classic definition of hip fractures, including femoral head, acetabular, or pubic fractures. Although our database does not permit direct assessment of these biases, a nationwide study in Uruguay reported a misclassification rate of 6.3% using ICD-10 (27).

Another potential reason for the lack of surgery is intrahospital mortality; however, a study from Northern Ireland indicated that intrahospital mortality was a barrier to surgical intervention for fewer than 1% of patients over a 15-year period (28). In Chile, a study conducted at a public hospital in Viña del Mar reported an intrahospital mortality rate of 3.09%, with 80% of the patients not being offered surgery (29). Barahona et al. reported a similar 3.35% intrahospital mortality rate among patients aged 60 and older in 2017, based on data from the National Health Ministry (13). Hence, we estimate that the attributable weight of intrahospital mortality to non-surgical treatment in Chile is about 2.7% (80% of 3.35%).

Considering both the 2.7% intrahospital mortality rate and the 6.3% misclassification rate, not more than 9% of non-surgical patients can be explained. Despite this adjustment, the figure remains significant and raises concerns regarding the management of hip fractures in Chile. Referring back to the study reporting an 18% rate of non-surgical treatment at a public hospital in Viña del Mar, the decision against surgery was influenced by a 30-day mortality risk of 20% in 20% of cases. Furthermore, in 30% of cases, the decision was based on the expectation of minimal functional improvement, as indicated by the Barthel Index, and 50% of patients met both criteria (15). Additionally, the non-surgical cohort had a median of 6 comorbidities, compared to 5 in the surgical cohort (15), a statistically significant difference but, in our opinion, not clinically relevant. Rutenberg et al. (30) demonstrated that, even among patients at high surgical risk, undergoing surgery reduced one-year mortality from 0.67 in the non-operated cohort (11% of the total cohort) to 0.48 in the operated cohort.

Unfortunately, other reports from South America highlight two-digit rates of non-surgical treatment for hip fractures. In Colombia, a university hospital reported a 31% non-surgical rate due to factors such as intrahospital mortality, high surgical risk, and financial barriers to surgery (31). In Peru, a hospital in Lima reported a 16% intrahospital mortality rate for patients awaiting surgery, often due to complications like pressure ulcers (32). Similarly, high non-surgical rates are reported in the other two studies (33, 34), which echo the 2021 Global Monitoring Report's note of a widespread lack of access to essential health services (35). Moreover, high non-surgical rates are often underreported because poor outcomes rarely motivate publication of such findings.

The demographics in our study, particularly age and gender, align with other research and do not explain the disparities in surgical access or hospital stay lengths. For instance, studies from Northern Ireland (28) and France (3) report similar median ages and hospital stays, but shorter surgery wait times than in Chile. There are also positive experiences in South America. An Argentine hospital reported a median hospital stay of seven days for patients over 85, with a one-year mortality rate of 24% (36).

The disparities we observed must be interpreted in the context of structural differences between the public and private healthcare sectors**,** particularly in the organisation and utilisation of operating rooms. Although our dataset does not directly measure operating room allocation or surgical delay, the marked differences in access to surgery and survival strongly suggest that inefficiencies in public operating room use—such as reliance on elective theatres with restricted schedules—play a central role. In private hospitals, surgical procedures are compensated based on performance, whereas in public hospitals, they are scheduled according to a fixed schedule. This fundamental distinction has been shown to significantly reduce hospital stays and one-year mortality rates at a national level (37). Furthermore, elective operating rooms in public hospitals are structured and allocated to various surgical specialities, typically from 8 a.m. to 5 p.m., and are inefficient, with only two surgeries per working day (9, 38). Consequently, their availability remains constant, irrespective of the number of patients awaiting hip surgery. In contrast, private institutions provide 24/7 on-demand access to operating rooms.

Consequently, the bottleneck in operating room availability leads to prolonged hospital stays. Supporting this interpretation, a recent neural network analysis from a Chilean public hospital demonstrated that waiting time for surgery is the primary determinant of extended hospitalisation, rather than patient comorbidity profiles (39). Patients remain uncertain while awaiting an available operating room, creating a cycle in which complications arising from the extended wait period sometimes render surgery riskier or even unfeasible (29, 40, 41). A recent study conducted in a public hospital in the capital of Chile reported that patients who underwent surgery after a waiting period of seven days had a one-year mortality rate of 39%, compared to a 20% rate for those operated on within seven days (42). Another study from a university hospital in Chile found that patients operated on after five days exhibited diminished survival compared to those who underwent surgery within five days, with follow-up extending to five years (43). The literature consistently shows that early surgery improves 30-day, one-year, and long-term survival rates (44). Minimising surgical delays while managing comorbidities is crucial (45). Furthermore, streamlined institutional models are cost-effective in managing hip fractures (46, 47).

Another critical issue is the availability of surgeons (48). In 2020, Chile had 1,778 certified orthopaedic surgeons, about one per thousand inhabitants (49). Chilean hip surgeons favour total hip arthroplasty (THA) for hip fractures, with usage rates of around two-thirds (50), a trend similar to that in the United States (51). In contrast, Australia (52) and the UK (53) have reduced THA rates (24% and 50%, respectively), opting instead for partial hip arthroplasty, which general trauma surgeons can perform. This allows for earlier surgeries without requiring a specialised hip surgeon for every case. While THA generally offers better prosthesis survival, the median age of hip fracture patients is 80, and their survival rates are lower compared to those of osteoarthritis patients (54) and the general population, as found in the present study. Additionally, partial hip arthroplasty is associated with lower costs and shorter surgical times, making it a significant consideration for healthcare policy (54).

Case volume also stresses the system (48), with over 90% of hip fractures treated in public hospitals. Preventing fractures is key to reducing their burden on healthcare (55). Although our study shows a stable age-adjusted incidence, Chile's ageing population is likely to increase the absolute number of cases (56). To reduce the incidence of hip fractures, it is essential to identify high-risk populations for sarcopenia and osteoporosis and implement effective fall prevention strategies (57–61).

The three critical non-modifiable factors contributing to elevated mortality rates emphasise the need for better policies. In the short term, allocating at least one 24/7 operating room per region specifically for fracture surgeries would expedite surgeries and reduce reliance on elective operating rooms. Furthermore, an interdisciplinary approach to hip fracture care is essential to improve access to timely surgery and shorten hospital stays (62, 63). While increasing transfers to the private sector may help, this practice should be minimised once dedicated operating rooms for the management of fractures are implemented.

A national registry is crucial for tracking outcomes and improving care (64). Other countries have seen significant improvements through clinical auditing and outcome monitoring (4, 46, 65). A registry would allow Chile to address specific regional and institutional challenges by generating localised data and enhancing care nationwide. This study, which incorporates data science, acknowledges the limitations of generalised data and highlights the need for localised insights to capture the unique circumstances of each healthcare setting. Chile has a long-standing success in maintaining registries for infectious diseases and vaccinations (66, 67), including the rapid inclusion of COVID-19 (68). This demonstrates the feasibility of extending this approach to other pathologies, such as hip fractures.

While our findings support the urgent need to optimise operating room allocation and the overall management of hip fracture surgery in Chile, it must also be recognised that, even under optimal conditions, survival after hip fracture remains lower than that of the general population. Therefore, equal emphasis should be placed on prevention strategies—such as osteoporosis management, fall prevention programs, and early identification of high-risk patients—since reducing the incidence of hip fractures remains the most effective way to diminish their long-term mortality burden.

This study has several strengths and makes novel contributions to the Chilean and international literature. First, it covers a six-year period (2012–2017), providing a broader temporal perspective than earlier reports that focused on single-year analyses (13). Second, it integrates two complementary national databases—hospital discharge records and death registries—allowing for robust ascertainment of both incidence and survival outcomes. Third, we estimated age- and sex-adjusted incidence rates, which provide a more accurate epidemiological picture than previous studies (12) and enable direct comparison with international benchmarks. Fourth, we applied survival analysis using Cox proportional hazards models, which enabled us to assess long-term mortality risk and adjust for covariates, thereby strengthening the causal interpretation. Fifth, by explicitly comparing outcomes between the public and private sectors, this is the first Chilean study to link disparities to inefficiencies in operating room management and to provide a concrete policy recommendation: the allocation of dedicated operating rooms for fracture surgery. Finally, our work situates Chile within both OECD countries with strong public health systems (e.g., France, the UK, Australia, and Northern Ireland) and Latin American neighbours (Peru, Colombia, and Argentina), thereby contextualising Chile's disparities in both global and regional terms.

This study has inherent limitations related to the use of administrative national databases, which may contain inaccuracies. However, cross-referencing with institutional records helped mitigate misclassification biases. Although the dataset does not include the exact time to surgery, a critical determinant of hip fracture outcomes, prolonged hospitalisation strongly reflects delays in surgical access in Chile. This interpretation is supported by previous national research showing that waiting time for surgery is the primary driver of extended length of stay rather than comorbidity burden (39). Importantly, although length of stay may partially reflect clinical complexity in selected cases, this factor alone is unlikely to account for the systematic and widespread delays observed. Moreover, frailer patients may benefit most from timely surgery once medically stabilised, as prolonged immobilisation while awaiting surgery can exacerbate existing comorbidities. At the population level, prolonged hospitalisation in this context more plausibly reflects organisational constraints, which may disproportionately harm frail patients by extending periods of preoperative immobilisation.

Socioeconomic status also influences survival after hip fracture, since life expectancy is closely linked to income. We addressed this by stratifying patients by FONASA tier, which directly reflects income. Importantly, we also compared outcomes among FONASA beneficiaries treated in public vs. private hospitals. Even though some residual socioeconomic confounding may persist, in many cases, public hospitals outsource patients to private clinics when demand exceeds capacity. In these situations, the patients themselves incur no additional costs. Finally, Comorbidity severity could not be directly assessed using validated indices such as the Charlson Comorbidity Index (CCI) or the ASA score due to database constraints. However, we incorporated prior hospitalisations (overall and cause-specific) as a pragmatic proxy for comorbidity burden, which demonstrated strong prognostic relevance in our analysis. Taken together, these findings reinforce that direct financial barriers do not explain the disparities we observed; rather, they are closely related to organisational inefficiencies in the delivery of surgical care.

## 5 Conclusions

In Chile, the crude incidence rate of hip fractures among individuals aged 60 and older remained constant from 2012 to 2017, with more than 90% of these cases receiving care within the public healthcare system. However, treatment in public hospitals is characterised by more extended hospital stays and restricted access to surgery compared to private hospital care, which can adversely affect patient survival.

The likelihood of mortality following a hip fracture is significantly influenced by modifiable factors, including access to surgical treatment, receiving care in public healthcare facilities, and the duration of hospitalisation. Additionally, non-modifiable factors such as age, male gender, and previous hospitalisations also play a crucial role.

In this context, optimising operating room allocation emerges as a key opportunity for improvement. We propose implementing pilot trauma-dedicated emergency operating rooms, prioritised in regions with the highest hip fracture incidence and surgical demand. Such targeted interventions could expedite surgical care, reduce prolonged hospitalisation, and ultimately improve survival outcomes, while informing scalable national policies.

## Data Availability

The databases used in this study are publicly available on the Department of Statistics and Health Information of Chile website (https://deis.minsal.cl/#datosabiertos).

## References

[1] Jayasekera PT Fernandopulle R Weerasenghe T de Soysa S Ranaweera T Edirisinghe E. Hip fractures and outcome in elderly patients in a tertiary care hospital of Sri Lanka. Arch Osteoporos. (2023) 18(1):113. 10.1007/s11657-023-01323-w 37672198

[2] Kelly M. Implementing findings from (hip) fracture registries. Injury. (2023) 54(Suppl 5):110961. 10.1016/j.injury.2023.110961 37563044

[3] Boukebous B Gao F Biau D. Hip fractures after 60 years of age in France in 2005–2017: nationwide sample of statutory-health-insurance beneficiaries. Orthop Traumatol Surg Res. (2023) 109(7):103677. 10.1016/j.otsr.2023.103677 37678611

[4] Neuburger J Currie C Wakeman R Tsang C Plant F De Stavola B The impact of a national clinician-led audit initiative on care and mortality after hip fracture in England: an external evaluation using time trends in non-audit data. Med Care. (2015) 53(8):686–91. 10.1097/MLR.0000000000000383 26172938 PMC4501693

[5] National Clinical Guidelines Centre. Update of the Management of Hip Fracture in Adults. London, National Institute for Health and Care Excellence (NICE) (2023). Available online at: https://www.nice.org.uk/guidance/cg124 (Accessed December 5, 2023).

[6] Peng K Yang M Tian M Chen M Zhang J Wu X Cost-effectiveness of a multidisciplinary co-management program for the older hip fracture patients in Beijing. Osteoporos Int. (2020) 31(8):1545–53. 10.1007/s00198-020-05393-1 32219498

[7] Fondo Nacional de Salud de Chile (FONASA). Reporte Anual De Fonasa (2019). Available online at: https://www.fonasa.cl/sites/fonasa/documentos (Accessed December 5, 2023).

[8] Ministerio de Salud de Chile. Manual Series Rem 2021–2022 (2021). Available online at: https://estadistica.ssmso.cl/download/manual-series-rem-2021-2022 (Accessed November 12, 2022).

[9] Barahona M Cárcamo M Barahona M Barrientos C Infante C Martínez Á. Estimación de la eficiencia del uso de pabellones electivos en el sistema de salud público chileno entre 2018 Y 2021. Medwave. (2023) 23(3):e2667. 10.5867/medwave.2023.03.2667 37011148

[10] Barahona M Cárcamo M Barrientos C Infante C Martínez Á. Access to knee arthroplasty among national health fund beneficiaries in Chile between 2004 and 2021. Medwave. (2023) 23(1):e2668. 10.5867/medwave.2023.01.2668 36720104

[11] Fraser B. Violent protests in Chile linked to healthcare inequities. Lancet. (2019) 394(10210):1697–8. 10.1016/s0140-6736(19)32720-5 31709990

[12] Diaz-Ledezma C Bengoa F Dabed D Rojas N López A. Hip fractures in the elderly Chilean population: a projection for 2030. Arch Osteoporos. (2020) 15(1):116. 10.1007/s11657-020-00794-5 32720199

[13] Barahona M Martínez Á Brañes J Rodríguez D Barrientos C. Incidencia, factores de riesgo Y letalidad de la fractura de cadera en Chile: estudio transversal sobre registros nacionales de 2017. Medwave. (2020) 20(5):e7939. 10.5867/medwave.2020.05.7939 32603321

[14] Zamora T Klaber I Bengoa F Botello E Schweitzer D Amenábar P. Controversias en el manejo de la fractura de cadera en el adulto mayor. Encuesta nacional a traumatólogos especialistas en cirugía de cadera. Rev Med Chil. (2019) 147(2):199–205. 10.4067/s0034-98872019000200199 31095168

[15] Dinamarca-Montecinos JL Améstica-Lazcano G Rubio-Herrera R Carrasco-Buvinic A Vásquez A. Características epidemiológicas Y clínicas de las fracturas de cadera en adultos mayores en un hospital público chileno. Rev Med Chil. (2015) 143(12):1552–9. 10.4067/S0034-98872015001200008 26928617

[16] Ahmad OB Boschi-Pinto C Lopez AD Murray CJ Lozano R Inoue M. Age Standardization of Rates: A New Who Standard. Geneva: World Health Organisation (2001), 9(10):1–14. Available online at: https://api.semanticscholar.org/CorpusID:5431703 (Accessed December 5, 2023).

[17] Ministerio de Salud de Chile. Encuesta De Caracterización Socioeconómica Nacional: Salud Casen En Pandemia (2020). Available online at: https://observatorio.ministeriodesarrollosocial.gob.cl/encuesta-casen-en-pandemia-2020 (Accessed January 15, 2024).

[18] Kannegaard PN van der Mark S Eiken P Abrahamsen B. Excess mortality in men compared with women following a hip fracture. National analysis of comedications, comorbidity and survival. Age Ageing. (2010) 39(2):203–9. 10.1093/ageing/afp221 20075035

[19] Riska BSL Forsén L Omsland TK Søgaard AJ Meyer HE Holvik K. Does the association of comorbidity with 1-year mortality after hip fracture differ according to gender? The Norwegian epidemiologic osteoporosis studies (norepos). J Am Geriatr Soc. (2018) 66(3):553–8. 10.1111/jgs.15207 29427505

[20] Hsu I-L Chang C-M Yang D-C Chang Y-H Li C-C Hu SC Socioeconomic inequality in one-year mortality of elderly people with hip fracture in Taiwan. Int J Environ Res Public Health. (2018) 15(2):352. 10.3390/ijerph15020352 29462914 PMC5858421

[21] Veronese N Maggi S. Epidemiology and social costs of hip fracture. Injury. (2018) 49(8):1458–60. 10.1016/j.injury.2018.04.015 29699731

[22] Auais M Al-Zoubi F Matheson A Brown K Magaziner J French SD. Understanding the role of social factors in recovery after hip fractures: a structured scoping review. Health Soc Care Community. (2019) 27(6):1375–87. 10.1111/hsc.12830 31446636 PMC7039329

[23] Alnemer MS Kotliar KE Neuhaus V Pape H-C Ciritsis BD. Cost-effectiveness analysis of surgical proximal femur fracture prevention in elderly: a markov cohort simulation model. Cost Eff Resour Alloc. (2023) 21(1):77. 10.1186/s12962-023-00482-4 37880692 PMC10601292

[24] Australian Commission on Safety and Quality of Health. Hip Fracture Clinical Care Standard (2023). Available online at: https://www.safetyandquality.gov.au/standards/clinical-care-standards/hip-fracture-clinical-care-standard (Accessed December 5, 2023).

[25] Aprato A Cipolla A D'Amelio A Vergano LB Giaretta S Massè A. Isolated greater trochanter fractures. Acta Biomed. (2023) 94(S2):e2023094. 10.23750/abm.v94iS2.13815 37366186

[26] Mysliborski T. Injury of the femoral nerve associated with avulsion fracture of the lesser trochanter in athlete. Chir Narzadow Ruchu Ortop Pol. (1952) 17(4):335–8. PMID: . 13042912

[27] Sosa González AM Bruno Lamorte CM Fernández de la Vega V Mazzilli Hernández D Rey C Taibo Brancato LP Análisis epidemiológico multicéntrico de las fracturas de cadera en uruguay: importancia Y planificación de un registro nacional. An Fac Med. (2021) 8(2):e203. 10.25184/anfamed2021v8n2a10

[28] Tucker A Donnelly K McDonald S Craig J Foster A Acton J. The changing face of fractures of the hip in Northern Ireland: a 15-year review. Bone Joint J. (2017) 99-B(9):1223–31. 10.1302/0301-620X.99B9.BJJ-2016-1284.R1 28860404

[29] Montecinos JLD Lazcano GA Buvinic AJC. Mortalidad intrahospitalaria en adultos mayores chilenos con fractura de cadera: incidencia, causas Y otros elementos de interés. Rev Chil Ortop Traumatol. (2018) 59(02):041–6. 10.1055/s-0038-1668593

[30] Rutenberg TF Assaly A Vitenberg M Shemesh S Burg A Haviv B Outcome of non-surgical treatment of proximal femur fractures in the fragile elderly population. Injury. (2019) 50(7):1347–52. 10.1016/j.injury.2019.05.022 31142435

[31] Martinez Rondanelli A. Fracturas de cadera en ancianos: pronóstico, epidemiología. Aspectos generales: experiencia. Rev Colomb Ortop Traumatol. (2005) 19:20–8. Available online at: https://pesquisa.bvsalud.org/portal/resource/pt/lil-619240 (Accessed September 22, 2024).

[32] Cervantes Chipa R. Complicaciones De La Fractura De Cadera Asociada a La Mortalidad Durante La Pandemia De Covid-19 Del Hospital Nacional Edgardo Rebagliati Martins, 2020. Repositorio Universidad San Juan Bautista (2022). Available online at: https://repositorio.upsjb.edu.pe/item/04e0741c-eb2b-48b9-9376-e712e53a3565 (Accessed September 22, 2024).

[33] Palma Vásquez NE. Características Epidemiológicas De Fracturas Extracapsulares De Cadera. Repositorio Universidad San Martín de Porres (2015). Available online at: https://repositorio.usmp.edu.pe/handle/20.500.12727/1348 (Accessed September 22, 2024).

[34] Venero Vargas G. Factores Asociados a Morbimortalidad En Pacientes Geriátricos Hospitalizados Por Fractura De Cadera En El Hospital Nacional Adolfo Guevara Velasco, Cusco-2018. Repositorio de la Universidad San Antonio Abad del Cuzco (2019). Available online at: https://repositorio.unsaac.edu.pe/handle/20.500.12918/3990 (Accessed September 22, 2024).

[35] Achrekar A Akselrod S Barron GC Charles MA Clark H Dain K Universal health coverage is fundamental to preparing for a healthier and better tomorrow. Lancet Glob Health. (2024) 12(2):e190–1. 10.1016/S2214-109X(23)00591-0 38096889

[36] Benchimol J Elizondo C Giunta D Schapira M Pollan J Barla J Survival and functionality in the elderly over 85 years of age with hip fracture. Rev Esp Cir Ortop Traumatol. (2020) 64(4):265–71. 10.1016/j.recot.2020.02.001 32247622

[37] Metcalfe D Zogg C Judge A Perry D Gabbe B Willett K Pay for performance and hip fracture outcomes: an interrupted time series and difference-in-differences analysis in England and Scotland. Bone Joint J. (2019) 101-B(8):1015–23. 10.1302/0301-620X.101B8.BJJ-2019-0173.R1 31362544 PMC6683232

[38] Aguilar-Barrientos R Velasco C. ¿Cómo Se Usan Los Pabellones Quirúrgicos En Chile? Radiografía Al Sector Estatal De Salud. Documentos de trabajo N° 1. Instituto de Políticas Públicas de Salud (IPSUSS), Universidad San Sebastián (2022). Available online at: https://www.ipsuss.cl/ipsuss/site/docs/20221125/20221125010103/uso_pabellones_en_chile__2022__aguilar__velasco__ipsuss_.pdf (Accessed November 12, 2022).

[39] Diaz-Ledezma C Mardones R. Predicting prolonged hospital stays in elderly patients with hip fractures managed during the COVID-19 pandemic in Chile: an artificial neural networks study. HSS J. (2023) 19(2):205–9. 10.1177/15563316221120582 37051613 PMC9434193

[40] Armijo Jara JA. Factores Predisponentes a la Mortalidad En Postoperados De Fractura De Cadera En Adultos Mayores En El Hospital Nacional Guillermo Almenara Yrigoyen, 2019. Repositorio Universidad San Juan Bautista (2021). Available online at: https://repositorio.upsjb.edu.pe/item/2f1a851b-10c9-45fe-81a1-497805143f2f (Accessed September 22, 2024).

[41] Delgado NHF. Factores de Riesgo Asociados a Complicaciones Posquirúrgicas En Fracturas De Cadera En Pacientes Del Hospital Nacional Carlos Alberto Seguín Escobedo: Repositorio Universidad Nacional de San Agustín (2018). Available online at: http://repositorio.unsa.edu.pe/handle/UNSA/5535 (Accessed September 22, 2024).

[42] Guiloff R Valderrama C Edwards D Contreras M Vaisman A. Epidemiología Y mortalidad en pacientes con fractura de cadera: impacto de la latencia quirúrgica en una cohorte de pacientes de un hospital público en Chile. Rev Med Chil. (2023) 151(11):1456–63. 10.4067/s0034-98872023001101456 39270112

[43] Barahona M Barrientos C Cavada G Brañes J Martinez Á Catalan J. Survival analysis after hip fracture: higher mortality than the general population and delayed surgery increases the risk at any time. Hip Int. (2020) 30(1_suppl):54–8. 10.1177/1120700020938029 32907421

[44] Beaupre L Khong H Smith C Kang S Evens L Jaiswal P The impact of time to surgery after hip fracture on mortality at 30-and 90-days: does a single benchmark apply to all? Injury. (2019) 50(4):950–5. 10.1016/j.injury.2019.03.031 30948037

[45] Vrahas MS Sax HC. Timing of operations and outcomes for patients with hip fracture—it’s probably not worth the wait. JAMA. (2017) 318(20):1981–2. 10.1001/jama.2017.17624 29183051

[46] Sahota O Currie C. Hip fracture care: all change. Age Ageing. (2008) 37(2):128–9. 10.1093/ageing/afn007 18349006

[47] Pollmann CT Røtterud JH Gjertsen J-E Dahl FA Lenvik O Årøen A. Fast track hip fracture care and mortality–an observational study of 2230 patients. BMC Musculoskelet Disord. (2019) 20(1):248. 10.1186/s12891-019-2637-6 31122228 PMC6533651

[48] Okike K Chan PH Paxton EW. Effect of surgeon and hospital volume on morbidity and mortality after hip fracture. J Bone Joint Surg Am. (2017) 99(18):1547–53. 10.2106/JBJS.16.01133 28926384

[49] Barahona M de los Santos D Díaz N Barrientos C Infante CA. Trends in orthopedic surgery in Chile: analysis between 2004 and 2020. Cureus. (2021) 13(5):e15080. 10.7759/cureus.15080 34017670 PMC8129445

[50] Barahona M Barrientos C Escobar F Sr Diaz N Palma D Sr Barahona MA Trends in knee and hip arthroplasty in Chile between 2004 and 2019. Cureus. (2020) 12(12):e12185. 10.7759/cureus.12185 33364139 PMC7752776

[51] Hochfelder JP Khatib ON Glait SA Slover JD. Femoral neck fractures in New York state. Is the rate of tha increasing, and do race or payer influence decision making? J Orthop Trauma. (2014) 28(7):422–6. 10.1097/BOT.0000000000000037 24343251

[52] Harris I Cuthbert A de Steiger R Lewis P Graves S. Practice variation in total hip arthroplasty versus hemiarthroplasty for treatment of fractured neck of femur in Australia. Bone Joint J. (2019) 101-B(1):92–5. 10.1302/0301-620X.101B1.BJJ-2018-0666.R1 30601055

[53] Khan A Rafferty M Daurka J. Hemiarthroplasty compared with total hip arthroplasty in fractured neck of femur: a shift in national practice? Ann R Coll Surg Engl. (2019) 101(2):86–92. 10.1308/rcsann.2018.0123 30112942 PMC6351869

[54] Guyen O. Hemiarthroplasty or total hip arthroplasty in recent femoral neck fractures? Orthop Traumatol Surg Res. (2019) 105(1S):S95–S101. 10.1016/j.otsr.2018.04.034 30449680

[55] Salech F Marquez C Lera L Angel B Saguez R Albala C. Osteosarcopenia predicts falls, fractures, and mortality in Chilean community-dwelling older adults. J Am Med Dir Assoc. (2021) 22(4):853–8. 10.1016/j.jamda.2020.07.032 32921573

[56] Girgis CM Clifton-Bligh RJ. Osteoporosis in the age of COVID-19. Osteoporos Int. (2020) 31(7):1189–91. 10.1007/s00198-020-05413-0 32346775 PMC7187664

[57] Yu F Xia W. The epidemiology of osteoporosis, associated fragility fractures, and management gap in China. Arch Osteoporos. (2019) 14(1):32. 10.1007/s11657-018-0549-y 30848398

[58] Papadimitriou N Tsilidis KK Orfanos P Benetou V Ntzani EE Soerjomataram I Burden of hip fracture using disability-adjusted life-years: a pooled analysis of prospective cohorts in the chances consortium. Lancet Public Health. (2017) 2(5):e239–e46. 10.1016/S2468-2667(17)30046-8 29253489

[59] Lamb SE Bruce J Hossain A Ji C Longo R Lall R Screening and intervention to prevent falls and fractures in older people. N Engl J Med. (2020) 383(19):1848–59. 10.1056/NEJMoa2001500 33211928

[60] Stathi A Greaves CJ Thompson JL Withall J Ladlow P Taylor G Effect of a physical activity and behaviour maintenance programme on functional mobility decline in older adults: the react (retirement in action) randomised controlled trial. Lancet Public Health. (2022) 7(4):e316–26. 10.1016/S2468-2667(22)00018-6 35325627 PMC8967718

[61] Pierrie SN Wally MK Churchill C Patt JC Seymour RB Karunakar MA. Pre-hip fracture falls: a missed opportunity for intervention. Geriatr Orthop Surg Rehabil. (2019) 10:2151459319856230. 10.1177/2151459319856230 31218094 PMC6558529

[62] Riemen AH Hutchison JD. The multidisciplinary management of hip fractures in older patients. Orthop Trauma. (2016) 30(2):117–22. 10.1016/j.mporth.2016.02.004 27418950 PMC4921687

[63] Shenouda M Silk Z Radha S Bouanem E Radford W. The introduction of a multidisciplinary hip fracture pathway to optimise patient care and reduce mortality: a prospective audit of 161 patients. Open Orthop J. (2017) 11:309. 10.2174/1874325001711010309 28567160 PMC5420169

[64] Sáez-López P Brañas F Sánchez-Hernández N Alonso-García N González-Montalvo J. Hip fracture registries: utility, description, and comparison. Osteoporos Int. (2017) 28(4):1157–66. 10.1007/s00198-016-3833-5 27872956

[65] Currie CT Hutchison JD. Audit, guidelines and standards: clinical governance for hip fracture care in Scotland. Disabil Rehabil. (2005) 27(18-19):1099–105. 10.1080/09638280500030475 16278178

[66] Wolff M. Cambios epidemiológicos en las enfermedades infecciosas en Chile durante la década 1990–2000: 1990–2000. Rev Med Chil. (2002) 130(4):353–62. 10.4067/S0034-98872002000400008 12090099

[67] Saldaña A Rodríguez M Roldán J Lobos C González C Avendaño M Farmacovigilancia de vacunas Y su aplicación en Chile. Rev Med Clin Condes. (2020) 31(3):240–55. 10.1016/j.rmclc.2020.05.005

[68] Taramasco C Rimassa C Romo JA Zavando AC Bravo RF. Epidemiological surveillance during the COVID-19 pandemic. The epivigila system. Medwave. (2022) 22(5):e8741. 10.5867/medwave.2022.05.8741 35667029

