# Supplementary material for: Disparities in survival after hip fractures in Chile in patients above 60 years: the impact of the operating room management
Source: Front Health Serv. 2026 Mar 13;6:1554376. doi: 10.3389/frhs.2026.1554376 (PMC13021676; doi:10.3389/frhs.2026.1554376)
Supplement: Supplementary file 1 [file Table1.docx]

|  | | **2012** | **2013** | **2014** | **2015** | **2016** | **2017** | **Total** |
| --- | --- | --- | --- | --- | --- | --- | --- | --- |
| Number of hip fractures | | 5,179 | 5,206 | 5,156 | 5,592 | 5,803 | 6,196 | 33,132 |
| Number of hip surgeries (% surgeries from total hip fractures) | | 3,896 (75%) | 3,885 (75%) | 3,944 (76%) | 4,280 (77%) | 4,475 (77%) | 4,813 (78%) | 25,293 (76%) |
| Type of hospital-health insurance provider | |  | | | | | | |
| Public hospital-Fonasa | # Hip fractures | 4,314 | 4,409 | 4,281 | 4,616 | 4,762 | 5,127 | 27,509 |
|  | # Surgeries (% from hip fractures) | 3,205 (74%) | 3,177 (72%) | 3,157 (74%) | 3,423 (74%) | 3,559 (75%) | 3,877 (76%) | 20,398 (74%) |
| Public hospital-Isapre | # Hip fractures | 47 | 19 | 15 | 45 | 14 | 21 | 161 |
|  | # Surgeries (% from hip fractures) | 35 (74%) | 15 (79%) | 10 (67%) | 37 (82%) | 11 (79%) | 13 (62%) | 121 (75%) |
| Public hospital - AF/NP & Others | # Hip fractures | 63 | 57 | 54 | 78 | 44 | 57 | 353 |
|  | # Surgeries (% from hip fractures) | 51 (81%) | 46 (81%) | 36 (67%) | 54 (69%) | 31 (70%) | 37 (65%) | 255 (72%) |
| Private hospital -Fonasa | # Hip fractures | 254 | 237 | 293 | 292 | 314 | 311 | 1,701 |
|  | # Surgeries (% from hip fractures) | 218 (86%) | 208 (88%) | 279 (95%) | 261 (89%) | 283 (90%) | 279 (90%) | 1,528 (90%) |
| Private hospital -Isapre | # Hip fractures | 193 | 208 | 240 | 283 | 315 | 365 | 1,604 |
|  | # Surgeries (% from hip fractures) | 165 (85%) | 195 (94%) | 219 (91%) | 264 (93%) | 293 (93%) | 349 (96%) | 1,485 (93%) |
| Private hospital – AF/NP & Others | # Hip fractures | 308 | 276 | 273 | 278 | 354 | 315 | 1,804 |
|  | # Surgeries (% from hip fractures) | 222 (72%) | 244 (88%) | 243 (89%) | 241 (87%) | 298 (84%) | 258 (82%) | 1,506 (83%) |
| Sex | |  | | | | | | |
| Male hip fractures (%) | | 1,161 (22%) | 1,228 (24%) | 1,172 (23%) | 1,249 (22%) | 1,349 (23%) | 1,375 (22%) | 7,534 (23%) |
| Female hip fractures (%) | | 4,018 (78%) | 3,978 (76%) | 3,984 (77%) | 4,343 (78%) | 4,454 (77%) | 4,821 (78%) | 25,598 (77%) |
| Use of public hospitals by type of insurance provider | |  | | | | | | |
| Isapre hip fractures (%) | | 47 (20%) | 19 (8%) | 15 (6%) | 45 (14%) | 14 (4%) | 21 (5%) | 161 (9%) |
| Fonasa D hip fractures (%) | | 325 (70%) | 293 (68%) | 262 (57%) | 312 (62%) | 331 (62%) | 376 (64%) | 1,899 (63%) |
| Fonasa C hip fractures (%) | | 357 (87%) | 336 (91%) | 241 (94%) | 256 (93%) | 328 (96%) | 339 (94%) | 1,857 (92%) |
| Fonasa B hip fractures (%) | | 2,628 (98%) | 3,109 (98%) | 3,229 (98%) | 3,547 (98%) | 3,581 (98%) | 4,043 (98%) | 20,137 (98%) |
| Fonasa A hip fractures (%) | | 1,004 (100%) | 669 (99%) | 549 (99%) | 501 (99%) | 522 (100%) | 369 (99%) | 3,614 (99%) |
| AF/NP hip fractures (%) | | 63 (17%) | 57 (17%) | 54 (17%) | 78 (22%) | 44 (11%) | 57 (15%) | 353 (16%) |
| Length of hospital stay in days (standard deviation) | |  |  |  |  |  |  |  |
| Overall | | 17.9 (17.7) | 18.1 (17.7) | 17.8 (16.0) | 18.4 (19.0) | 17.3 (17.2) | 16.7 (17.6) | 17.7 (17.6) |
| Public hospitals | | 19.1 (18.5) | 19.2 (18.3) | 18.9 (16.4) | 19.7 (19.8) | 18.6 (18.0) | 18.0 (18.5) | 18.9 (18.3) |
| Private hospitals | | 11.1 (9.7) | 11.3 (10.8) | 11.5 (11.3) | 11.0 (11.3) | 10.8 (10.6) | 10.0 (8.9) | 10.9 (10.4) |

**Supplementary Table 1. Annual distribution of hip fractures and surgical treatment among Chilean adults aged ≥60 years, by hospital type, health insurance provider, and sex, 2012–2017.**
The table presents the annual number of hip fractures and surgeries, disaggregated by hospital ownership (public or private), insurance type (FONASA categories A–D, ISAPRE, Armed Forces/National Police), and sex. Percentages represent the proportion of surgeries among total hip fractures within each subgroup. Additional sections show the utilisation of public hospitals by insurance category and the average length of hospital stay (mean ± standard deviation) by hospital type.
